# Gene Fitness Landscapes of *Vibrio cholerae* at Important Stages of Its Life Cycle

**DOI:** 10.1371/journal.ppat.1003800

**Published:** 2013-12-26

**Authors:** Heather D. Kamp, Bharathi Patimalla-Dipali, David W. Lazinski, Faith Wallace-Gadsden, Andrew Camilli

**Affiliations:** Howard Hughes Medical Institute and Department of Molecular Biology and Microbiology, Tufts University School of Medicine, Boston, Massachusetts, United States of America; University of Texas San Antonio, United States of America

## Abstract

*Vibrio cholerae* has evolved to adeptly transition between the human small intestine and aquatic environments, leading to water-borne spread and transmission of the lethal diarrheal disease cholera. Using a host model that mimics the pathology of human cholera, we applied high density transposon mutagenesis combined with massively parallel sequencing (Tn-seq) to determine the fitness contribution of >90% of all non-essential genes of *V. cholerae* both during host infection and dissemination. Targeted mutagenesis and validation of 35 genes confirmed our results for the selective conditions with a total false positive rate of 4%. We identified 165 genes never before implicated for roles in dissemination that reside within pathways controlling many metabolic, catabolic and protective processes, from which a central role for glycogen metabolism was revealed. We additionally identified 76 new pathogenicity factors and 414 putatively essential genes for *V. cholerae* growth. Our results provide a comprehensive framework for understanding the biology of *V. cholerae* as it colonizes the small intestine, elicits profuse secretory diarrhea, and disseminates into the aquatic environment.

## Author Summary

Cholera is a deadly diarrheal disease that spreads in explosive epidemics and is caused by the water-borne bacterium *Vibrio cholerae*. Pathogenic strains of *V. cholerae* can be found in both fresh and salt water estuaries in-between cholera outbreaks. Cholera infections are frequently derived from contaminated fresh water sources. In this study, we sought to determine on a genome-wide scale how *V. cholerae* is able to colonize and proliferate in the nutrient-rich environment of the small intestine, but then also survive dissemination and persist in the nutrient-limited aquatic environment. Using a host model that mimics the pathology of human cholera, we utilized genome-wide transposon mutagenesis and massively parallel sequencing of the insertion junctions to obtain the relative fitness of *V. cholerae* mutants during infection and dissemination. This extensive data set represents the first genetic screen of any kind to identify genes important for dissemination into the environment and has broad significance for understanding and controlling the spread and persistence of *Vibrio cholerae* and potentially other water-borne pathogens in the environment.

## Introduction

The human intestinal pathogen *Vibrio cholerae* is a natural inhabitant of the aquatic ecosystem. *V. cholerae* is a halophile that normally resides in coastal waters and brackish estuaries, however pathogenic strains can persist in bodies of fresh water for extended periods of time in cholera endemic areas [1]. Human consumption of *V. cholerae* in contaminated food and water leads to colonization of the small intestine, extensive bacterial multiplication, and cholera. Activation of the virulence gene cascade upon colonization leads to the production and secretion of the cholera toxin (CT) into the intestinal lumen (reviewed in [2]). CT induces the secretion of water, electrolytes and mucin into the bowel resulting in profuse secretory diarrhea, known as rice water stool (RWS) [3]. CT-induced diarrhea can cause death by dehydration within hours in the absence of aggressive rehydration therapy [4]. Diarrheal expulsion of *V. cholerae* from the host into the aquatic environment leads to dissemination and transmission of cholera, thus completing the pathogen life cycle. Host-passaged *V. cholerae* are an order of magnitude more infectious than *in vitro* grown bacteria, and this hyperinfectious state is maintained for a limited time in fresh water [5], [6]. Contamination of household food and/or water with hyperinfectious *V. cholerae* can lead to rapid spread of the disease and fuels epidemics [7], [8].

When RWS *V. cholerae* are shed into fresh water, the bacteria encounter a drastic drop in osmolarity, temperature and nutrient availability [6]. Transcriptional analysis of RWS *V. cholerae* transitioned into pond water revealed repression of protein synthesis and energy metabolism genes and induction of phosphate and nitrogen scavenging genes, consistent with low levels of carbon, phosphate and nitrogen in the pond [6]. However, the importance of these genes for dissemination was not examined further. Indeed, very little is known about the genetic factors important for *V. cholerae* survival in fresh water after host passage. Glycogen storage is one of the few processes described that enhance dissemination of *V. cholerae* [9]. Glycogen is a branched glucose polymer that accumulates in stationary phase and is used as a carbon and energy source under nutrient limiting conditions, such as the aquatic environment. Glycogen storage granules have been detected in *V. cholerae* isolated from RWS, and some of the glycogen storage and utilization genes have been determined to be important in dissemination and transmission [9].

A few studies have suggested that host-passaged *V. cholerae* exit the host in a conditioned state to better survive the transition into fresh water [10], [11]. The data suggest that these host-passaged *V. cholerae* are similar to bacteria in stationary phase [11], [12]. Until recently, a suitable animal model to study dissemination into the aquatic environment was lacking. Although *V. cholerae* colonize the small intestine of the infant mouse, CT-induced secretory diarrhea is not produced. An infant rabbit model of infection was recently improved to give consistent and reproducible symptoms of secretory diarrhea that more closely resemble human disease [13]. In this study, we utilized the infant rabbit model of cholera to study both pathogenesis and subsequent dissemination into the aquatic environment. We sought to determine on a genome-wide basis the genetic determinants necessary for survival in both conditions. Using the Tn-seq method [14], [15], which combines saturating transposon mutagenesis with massively parallel sequencing (MPS) of transposon junctions, we quantitatively measured the fitness of each gene during both infection of the host small intestine and dissemination to a fresh water environment.

## Results

### High throughput transposon sequencing to determine genome-wide fitness

**Figure 1:**
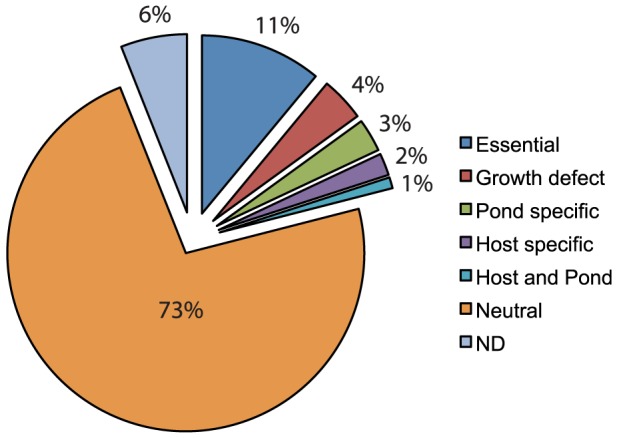
Pie chart representing the specific requirements for *V. cholerae* genes. This data represents fitness values calculated using Tn-seq on a near-saturating transposon insertion library used in 4 independent experiments, 3 different environmental conditions, and 9–21 biological replicates for each condition.

Eleven mini-Tn*5* (mTn*5*) transposon libraries were constructed in *V. cholerae* O1 serogroup, El Tor biotype strain E7946, representing over 100,000 unique transposon insertions as determined by MPS. From the Tn-seq data, we identified 414 genes that lacked Tn insertions, and therefore are deemed putatively essential for *V. cholerae* viability in rich medium (Figure 1 and Table S1). With 100,000 unique transposon insertions in the remaining 3471 non-essential genes (average of one insertion every 36 bp), our total library coverage is likely to be near saturating. Comparison of our essential gene list to those previously reported in *V. cholerae* C6706 [16], [17], showed 87% (361/414) overlap (Table S1). The libraries were tested in three selective conditions including rich medium (*in vitro*), host (*in vivo*), and dissemination from host to aquatic environment. The orogastric infant rabbit model of *V. cholerae* infection was used to represent the host selective condition [13], fresh pond water was used to represent the aquatic environment [6], [10] and Luria Bertani (LB) broth was used for the rich medium.

**Figure 2:**
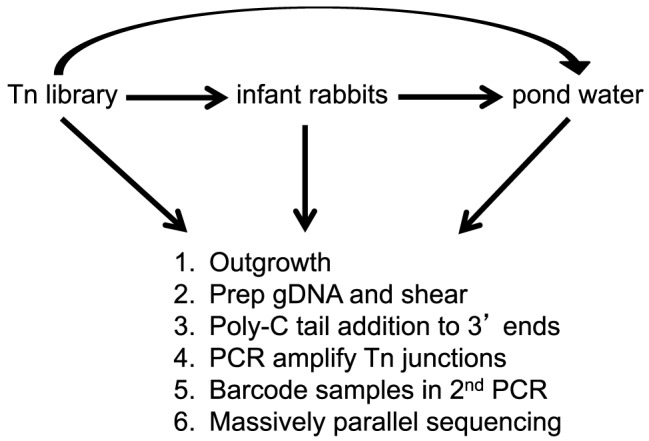
Flow diagram of experimental design. A library of mTn*5* transposon mutants of *V. cholerae* was used to infect infant rabbits. After 12 hrs of infection, the host-passaged *V. cholerae* were collected, pelleted, and resuspended in pond water for 48 hrs at 30°C. In addition, the library was also passaged in LB broth overnight and put directly into pond water for 48 hr at 30°C. After each selective condition the following steps were done: 1. Bacteria were minimally outgrown to a high titer in LB. 2. gDNA was prepared and sheared. 3. Poly-C tails were added to the 3′ ends using Terminal deoxynucleotidyl Transferase. 4. The transposon-genome junctions were amplified by PCR. 4. Barcodes were added to each sample by a second PCR. 5. Multiplexed samples were subjected to massively parallel sequencing.

After the transposon library was subjected to a selective condition (Figure 2), the surviving population was outgrown a limited number of generations in LB broth to achieve high cell density. This outgrowth eliminated the potential for DNA contamination from dead *V. cholerae* cells. Genomic DNA (gDNA) was isolated from each outgrown sample and processed as illustrated in Figure 2 (also see Experimental Procedures). The final PCR products contained sequences corresponding to the transposon, gDNA flanking the transposon, Illumina-specific sequences on each end necessary for MPS, and a unique 6 bp barcode embedded in one end to identify the sample. Samples were multiplexed and subjected to MPS to determine the frequency of each transposon insertion mutant in both the input and output populations.

The fitness contribution of each non-essential gene in the genome (hereafter referred to as “a gene's fitness”) was calculated for each selective condition as described [15], where each gene's fitness represents a measurement of the net growth (in LB broth or the host) or death (in pond) of that gene disruption relative to the bulk population. In order to be able to equate fitness with growth rate in the rich media and host conditions, we needed to determine the population expansion during each experiment. This is easily determined in LB broth by enumerating the total increase in colony-forming units (CFU), however bacterial growth is more difficult to assess during infection due to the stochastic and host-induced loss of bacteria during the course of infection. In order to determine the expansion of *V. cholerae* during host infection we utilized a low copy, temperature-sensitive plasmid that is replication defective at physiological temperatures [18]. As the bacteria divide and replicate *in vivo*, the temperature sensitive plasmid is lost by segregation [19]. After the course of host infection, we compare the plasmid positive (KanR) and plasmid negative (KanS) populations to determine the ratio of plasmid loss. By fitting the ratio of plasmid loss *in vivo* to an *in vitro* standard curve at physiological temperature, the expansion of the *in vivo* population was estimated and used in our fitness calculation.

Stochastic loss of bacteria due to narrow bottlenecks in the selective conditions can lead to noisy fitness calculations with large standard deviations. To widen the bottleneck in the *in vivo* selection, specifically during passage through the stomach, the following steps were taken; 1) we pre-conditioned *V. cholerae* by acid-tolerizing them for 1 hr prior to intragastric inoculation [20], 2) the inoculum was suspended in an alkaline bicarbonate-buffer, and 3) animals were pre-treated with Cimetidine to block stomach acid production as described [13]. The bottleneck, defined as the percentage of neutral genes lost stochastically, was never greater than 15% and the median ranged from 0–7% depending on the selective condition (Table 1).

**Table 1:** Summary of data analysis.

	Cecal fluid	Small Intestine	Cecal fluid→Pond	LB→Pond	LB→LB
Independent experiments	4	2	4	3	3
Biological replicates	16	10	21	12	9
Bottleneck* (median)	2%	5%	2%	7%	0%
Coverage#	93%	94%	90%	96%	94%
Significantly different than 1.0&	316	335	140	399	168
Different than 1.0 and LB→LB##	102	131	80	148	NA

^*^The bottleneck for each biological replicate is calculated by determining the number of insertion sites lost from the input to the output in a set of known neutral genes. The median bottleneck is reported.

^#^The coverage specifies the total number of genes for which fitness values were obtained as a percentage of the total number of non-essential genes in the *V. cholerae* genome.

^&^A one-sample student's t-test with Bonferroni correction on genes with ≥5 insertion sites was used to determine if the fitness value is statistically different than 1.0.

^##^A student t-test with Bonferroni correction on genes statistically different than 1.0 was used to determine if the fitness value obtained in the test condition is statistically different that the fitness value obtained in the LB grown culture. Additionally, we required at least a 0.15 fitness difference from LB and a fitness value in LB of at least 0.82.

To reduce the number of false positives and false negatives, the final fitness values of genes obtained under each selective condition were calculated from multiple insertion sites per gene (≥5) from 2–4 independent experiments representing a total of 9–21 biological replicates (Table 1). These stringent criteria resulted in exclusion of 6% of genes in LB that had less than 5 insertion sites but were not determined to be essential (11%) (Figure 1). Of the remaining 83% of genes, we were able to calculate fitness values in one or more selection conditions (Table 1 and Table S2). A fitness value of 1.0 is neutral, less than 1.0 is disadvantageous, and greater than 1.0 is advantageous. To determine if a fitness value was statistically different from 1.0, a one-sample student's t test with Bonferroni correction for multiple testing was used. To determine if a fitness value is specific for the selective condition, we required the fitness value to be statistically different than the fitness value obtained in LB and required at least a 0.15 fitness value difference between the two fitness values. In addition, any gene with a fitness value less than 0.82 in LB was considered to have a general growth defect and eliminated from the host and pond gene lists. These stringent requirements yielded between 80–148 genes required for each condition (Table 1 and Table S3). The percentage of genes within each class is shown in Figure 1.

### Survival in the host environment

**Figure 3:**
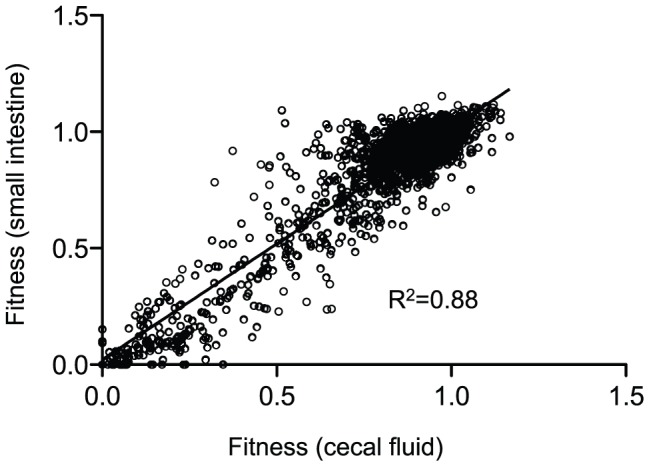
A correlation plot of fitness values from host-passaged *V. cholerae*. Each dot represents the fitness values for one gene in the two host compartments shown on the x- and y-axes. Only genes that had calculated fitness values in both conditions were plotted, which represents 93% of all non-essential genes in *V. cholerae*. A linear regression analysis was used to determine the correlation.

In contrast to the more commonly used infant mouse model, the orogastric model of infection in the infant rabbit better approximates severe human cholera because of the large volume of secretory diarrhea that results [13], [21]. Prior to diarrheal excretion, the luminal fluid and *V. cholerae* shed from the small intestine accumulate in the cecum of the infant rabbit [13]. Collection of the cecal fluid provided essentially pure cultures of host-passaged *V. cholerae* that was used to study both pathogenesis and dissemination into the aquatic environment. The fitness values calculated for genes obtained from the cecal fluid correlated well with those calculated using the small intestine (Figure 3), indicating that the former population is highly representative of the latter. Therefore, all genes identified as important for survival in the host were collectively determined by combining the statistically significant gene lists from both the cecal fluid and small intestine (Table 2 and Figure 1). There were 133 genes identified as being important for survival specifically in the host environment (Table 2 and Table S3). Over half of these genes (76 genes; 57%) were new factors that had not been previously implicated in *V. cholerae* infection, and thus the data set provides a wealth of information for future studies in *V. cholerae* pathogenesis. The remaining 57 genes (43%) had previously been shown to be important for *V. cholerae* colonization in the infant mouse model of infection, including the well-known virulence factors: ToxR, ToxS, ToxT, the TCP biosynthetic operon, and the lipopolysaccharide O-antigen biosynthetic operon (Table S3). Classification of the known or hypothesized functions of the genes important for survival reveals that a large number are required for purine and pyrimidine biosynthesis, O-antigen biosynthesis, and amino acid metabolism (Table 2 and Table S3). The remaining genes are spread across many functional classes including phosphate acquisition, post-translational modification, fatty acid metabolism, and transporters. Since the majority of the population has a neutral phenotype, some classes of mutations would be missed due to the ability of the bulk population to complement the mutant defect *in trans*. For example, the cholera toxin (CT) genes *ctxA* and *ctxB* had neutral fitness values since the remaining population secreting CT into the small intestine can complement their deletion *in trans*.

**Table 2:** Functional classification of genes important for infection and dissemination*.

Function	Host#	Pond$	Both%
Pathogenesis	15	0	2
purine/pyrimidine biosynthesis	14	2	2
amino acid metabolism and degradation	11	7	3
LPS/O-antigen biosynthesis	11	1	5
flagellar biosynthesis	0	15	3
Hypothetical	3	14	1
energy production/conversion	2	12	1
Transporter	6	11	5
cell wall/outer membrane biogenesis	2	10	1
transcriptional regulators/signal transduction	2	10	0
electron transport/cytochrome biogenesis	0	9	5
carbohydrate utilization	3	5	0
RNA modification	3	5	0
lipid/fatty acid metabolism	2	5	2
protein degradation	1	5	3
post-translational modification	4	4	0
co-enzyme utilization	0	3	2
translation factors	3	2	1
Miscellaneous	2	2	0
phosphate utilization	4	1	2
DNA modification and repair	2	1	0
glycogen storage and utilization	1	1	2
quorum sensing regulators	2	0	0
Total	93	125	40

^*^All the genes identified have been determined to be both significantly different that 1.0 and significantly different than LB. All of these genes and their fitness values are listed in Supplemental Table S3.

^#^Host factors include those genes identified in either small intestine or cecal fluid samples.

^$^Pond genes include those genes identified in either the LB→Pond or Cecal Fluid→Pond.

^%^Genes that are defective in Cecal Fluid and Cecal Fluid→Pond but are not defective in LB→Pond are counted as Host only.

### Dissemination into the aquatic environment

**Figure 4:**
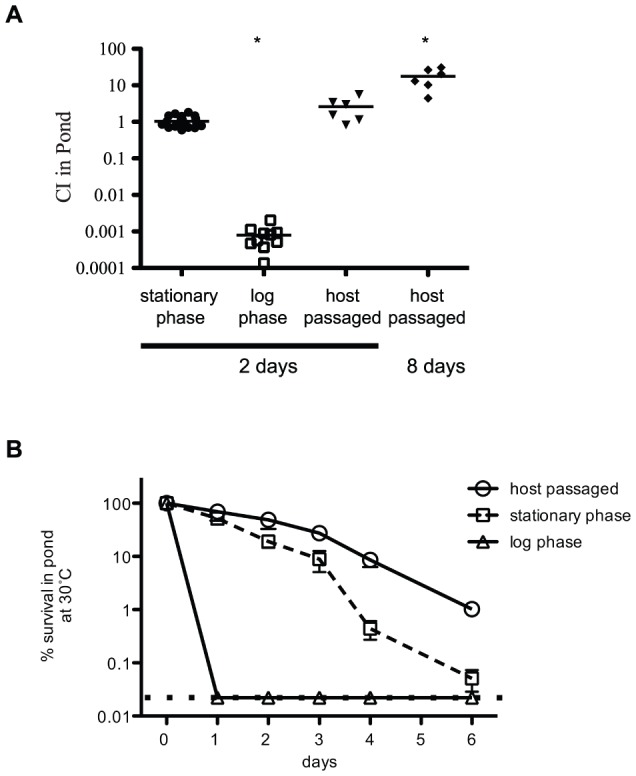
Growth-phase dependent survival of *V. cholerae* in pond water. (**A**) Competition assays in pond water. Either a wild type or a Δ*lacZ* strain were grown overnight to stationary phase and competed 1∶1 with the test strain in pond water for 2 or 8 days as indicated at 30°C. The first column is a control competition between the wild type and Δ*lacZ* stationary phase cells. Exponential-phase cultures were collected at OD_600_ = 0.3. Host passaged wild type was collected from the cecal fluid of infant rabbits 12 hrs post-infection. Each dot represents a biological replicate, and open squares indicate the limit of detection. Both the exponential phase (P value = <0.0001) and host-passaged (P value = 0.01) at 8 days are statistically different than 1.0. (**B**) Survival comparison in pond water. Stationary phase, exponential phase and host-passaged *V. cholerae* were pelleted, washed and resuspended in pond water. The pond cultures were placed standing at 30°C for 6 days and the CFU/ml was plated each day. At least 6 biological replicates were used for each time point. The input CFU at time zero was used to determine 100% survival. The dotted line indicates the limit of detection.

In addition to calculating genome-wide fitness values for host infection, we also determined fitness survival values for dissemination into the aquatic environment. We isolated host-passaged *V. cholerae* from the infant rabbit cecal fluid, which is similar in composition to human RWS [13]. From each infected animal, we obtained between 0.2–1.2 ml of cecal fluid containing ∼10^8^ CFU/ml of *V. cholerae*. These bacteria were pelleted, resuspended, and diluted into pond water and then incubated at 30°C for 2 days statically in an open container. Concurrently, transposon libraries grown in LB broth overnight to stationary phase were also pelleted, resuspended, and diluted into pond water. Direct comparison between host-passaged and LB stationary-phase bacteria using competition experiments on the bulk populations showed no significant difference in pond survival at 2 days (Figure 4A), however host-passaged *V. cholerae* show a significant survival advantage at 8 days (Mean competitive index [CI] = 18). In contrast to host-passaged and stationary phase bacteria, exponential growth-phase *V. cholerae* from LB broth do not survive well in pond water at 2 days (Figure 4). After 3 days in pond water, the survival of stationary phase bacteria also drops severely in contrast to host-passaged bacteria (Figure 4B). To avoid severe bottleneck restraints incurred at longer time points, we examined genome-wide fitness after 2 days post-dissemination of both host-passaged and stationary phase bacteria.

Statistical comparison to both the neutral fitness value of 1.0 and to the fitness value obtained in LB revealed 165 genes that are important for survival in the pond environment (Table 2 and Table S3). These included both host-passaged and stationary phase into pond selections (Table 2). Functional classification indicates that a large number of these genes have known or hypothetical roles in energy production and conservation, cell wall and outer membrane biogenesis, electron transport, flagellar biosynthesis, transcriptional regulation, transporters and hypothetical proteins. Since this is the first genome-wide screen to determine factors important for dissemination and survival in the aquatic environment, almost all of the genes identified are new.

### Validation of gene fitness values

**Figure 5:**
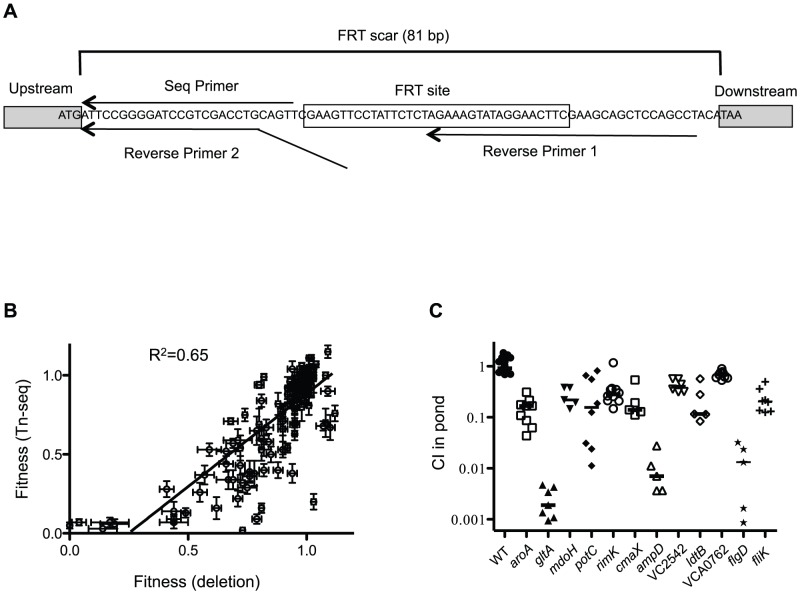
Validation of Tn-seq results by gene deletion. (**A**) Schematic of FRT scar use for high throughput sequencing. In-frame gene deletions were made using FRT/FLP recombination. Nested PCR using reverse primers 1 and 2 and a forward primer (not shown) was used to amplify the junction between the deletion scar and the gDNA. The sequencing primer is shown. (**B**) Correlation plot of fitness values of deletions compared to original Tn-seq screen. Each dot represents the fitness value and standard error (SE) for one gene in both screens shown on the x- and y-axes. Shown are paired fitness values from 35 gene deletions tested in 4 different conditions: LB, Cecal fluid, Small Intestine and Pond. A linear regression analysis was used to determine a correlation. (**C**) Validation of Frt-marked deletions in a 1∶1 competition with a Δ*lacZ* wild-type for 48 hr in pond water. Median values are indicated. Using a one sample t-test all CI values were significantly different than 1.0 (P≤0.001).

In order to validate the results of the Tn-seq screen, a mini-library of 35 gene deletions representing host-specific, pond-specific, neutral, and dual specificity factors covering a wide range of fitness values was constructed and tested collectively in the selective conditions (Table S4). This strategy allowed us to test a large number of genes while greatly reducing the number of animals needed for the experiment. These 35 deletions were constructed by combining allelic exchange by natural transformation with Frt/Flp recombination [22], resulting in an identical 81 bp in-frame “scar” between the start and stop codons in each deletion (Figure 5A). This 81 bp sequence flanks unique sequence at each deletion locus, with the latter serving as a barcode that can be used to track each mutant in the mini-library by MPS using an essentially identical protocol to the original Tn-seq screen (Figure 5A, Figure 2). In addition to re-testing these 35 deletion mutants in infant rabbits, pond, and rich media, we also infected infant mice with the mini-library to compare the two host models. For the infant mouse experiment, a CI was calculated based on MPS reads normalized to a neutral gene deletion in both the input and output pools. Further validation of the genes important for pond survival also included 1∶1 competitions in pond water with a wild-type strain (Figure 5C and Table S4).

Direct comparison between the fitness values obtained for all conditions in the Tn-seq screen to the fitness values obtained with the deletions revealed a strong correlation (Figure 5B); 97% (34/35) of the deletions were confirmed for the host selection, and 91% (32/35) of the deletions were confirmed for the pond selection (Table S4). The percentage of false positives are in line with 5% false discovery rate that was imposed in the Tn-seq statistical calculations [14]. Nine out of 11 infant rabbit host-specific factors were also shown to be important for virulence in the infant mouse (Table S4), suggesting that these two host environments are highly similar. The two factors that appear to be rabbit-specific are *gltA* and *mtlD*, encoding a citrate synthase and a mannitol metabolic protein, respectively. Interestingly, *gltA* is also important for survival in the aquatic environment (Figure 5C and Table S4). The necessity of these genes in the infant rabbit model but not the infant mouse model, suggests there are differences in carbon and energy sources available to *V. cholerae* in the two hosts.

**Figure 6:**
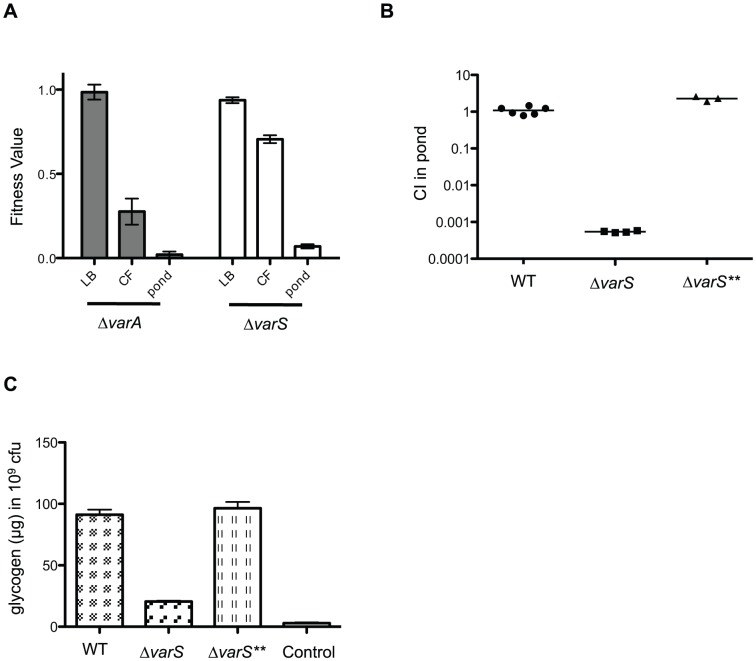
The VarS/VarA two component system regulates glycogen storage. (**A**) Fitness values obtained from the Tn-seq screen. The data represents the mean and standard error for each gene in rich media (LB), cecal fluid (CF), and pond. (**B**) Competition assay in pond water. A Frt-marked deletion of *varS* (Δ*varS*) and a suppressor strain (ΔvarS**) were competed 1∶1 with Δ*lacZ* wild-type for 48 hr in pond water. Median values are indicated. Using a one sample t-test the CI for Δ*varS* was significantly different than 1.0 (P≤0.0001). (**C**) Glycogen was measured in WT, Δ*varS*, and ΔvarS** after growth on Kornberg Media by enzymatic hydrolysis of glycogen into glucose monomers. Liberated glucose was subsequently measured using the tetrazolium blue reducing sugar assay. The mean and standard deviation of two independent experiments each performed in triplicate are shown. Using a two sample t test, the CI of Δ*varS* was significantly different than wild-type (<0.0001). The assay control was treated identically to wild-type but does not contain the enzymatic hydrolysis step and therefore measures the amount of background glucose present in WT cells.

The VarS sensor kinase and cognate VarA response regulator make up a two component system that regulates the transcription of virulence genes in *V. cholerae* [23], [24], [25] and controls both the expression and the activity of the HapR quorum sensing regulator [26], [27]. Both *varA* and *varS* deletion strains have colonization defects in mice [23], [25]. In our original screen, transposon insertions in both *varS* and *varA* resulted in fitness defects in both the host and in the pond, but not during growth in rich media (Figure 6A). To validate the pond phenotype we constructed a *varS* deletion strain and competed it 1∶1 with a wild-type strain in pond water for 48 hrs (Figure 6B). A Δ*varS* suppressor strain (ΔvarS**) which phenocopies the wild-type strain was isolated during the construction of Δ*varS*, due to nutrient-limited conditions required for natural transformation. Whole genome resequencing of ΔvarS** revealed a single nucleotide change located in the start codon (ATG→ATA) of *csrA* (carbon storage regulator gene A). However, there is a putative alternative start codon (TTG) 6 bp downstream with an appropriately spaced Shine-Dalgarno sequence, which we speculate provides reduced expression of CsrA. CsrA is a global post-transcriptional regulator that has been shown to control many processes in *E. coli* including glycogen synthesis and storage (Reviewed in [28]). The VarS/VarA homologs BarA/UvrY in *E. coli* antagonize CsrA by activating transcription of an inhibitory sRNA, *csrB*. The binding and sequestration of CsrA by *csrB* promotes translation of downstream targets, including the mRNA for the glycogen biosynthetic genes *glgCAP* [29]. Thus, the varS** suppressor is predicted to restore repression of CsrA thus allowing for expression of downstream CsrA-repressed genes. Since glycogen storage is known to be important for *V. cholerae* survival in the aquatic environment [9], we measured and compared the amount of glycogen in a wild-type strain of *V. cholerae* to both Δ*varS* and ΔvarS** (Figure 6C). The Δ*varS* strain has significantly less glycogen stored than both the wild-type strain and ΔvarS**, which likely accounts for the survival defect in the pond (Figure 6B).

## Discussion

The water-borne pathogen *V. cholerae* encounters disparate environmental conditions when it transitions between the human small intestine and the aquatic environment. Understanding the genetic determinants important for survival during each stage of the life cycle of this organism is key to understanding the dynamics of cholera outbreaks and persistence during and in between outbreaks. In this study, we determined genome-wide fitness in a mixed population for gene disruptions during both host infection and dissemination into a pond water aquatic environment. By using a host model that closely resembles human cholera, we have obtained new information about *V. cholerae* pathogenesis and for the first time have been able to identify factors important for dissemination from watery stools into the aquatic environment. Overall our study is unique in several respects: first, it presents the first genome-wide data set from a saturating mutant screen that identifies the roles of genes in a *V. cholerae* infection; second, it is the first genome-wide data set on genes important for dissemination from the host into an aquatic environment; and third, it represents the first genome-wide screen done using the infant rabbit model.

The current knowledge of *V. cholerae* virulence resides mostly in data obtained from a handful of non-saturating mutagenesis screens done using the infant mouse model of infection [20], [30], [31], [32]. While *V. cholerae* is able to colonize the small intestine of infant mice, it does not elicit the same fatal secretory diarrheal response as seen in human cholera; while however, this pathology is replicated in the infant rabbit host model [13]. A recent RNA-seq study comparing the *V. cholerae* transcriptome during infection of the infant mouse or infant rabbit found only partial overlap between the two hosts [33]. Despite the differences in *V. cholerae* gene expression and disease outcome between the two host models, we identify 57 virulence factors in the infant rabbit model that had previously been shown to be important in the infant mouse model. Among these previously identified factors are the genes required for TCP biogenesis, O-antigen synthesis, phosphate acquisition, quorum sensing regulators, and key virulence transcriptional regulators including ToxR, ToxS, ToxT, VarS, VarA and AphA. As part of the validation of our Tn-seq screen we compared the roles of 11 genes in colonization of the infant rabbit to that of the infant mouse. A majority of the genes (9/11), were important for colonization in both models. Thus, our data indicate that the requirements for infection of these two seemingly dissimilar animal hosts are remarkably comparable.

Among the notable differences in gene requirement in the two animal models are the well-known virulence factors TcpP, TcpH, and the accessory colonization factors AcfA-D, which gave mild or neutral phenotypes in the infant rabbit host. In the infant mouse model and in laboratory conditions that stimulate virulence gene expression, TcpPH is known to function with ToxRS to co-stimulate transcription of *toxT*, which encodes the main transcriptional activator of the ToxR virulence regulon [34], [35]. Interestingly, a recent study using the infant rabbit model showed that transcription of *tcpPH* is not induced during infection when compared to *in vitro* transcription [33]. The same study also revealed that the biosynthetic genes for vibriobactin, an iron siderophore important for infant mouse colonization, are not induced in the infant rabbit, and that instead heme may be used as the main iron source during infant rabbit colonization [33]. We confirm this hypothesis at the phenotypic level, by showing that vibriobactin genes are dispensable during infant rabbit colonization, while a heme transporter (VC2056) is needed.

It has been previously demonstrated that *V. cholerae* incorporates exogenous fatty acids from host bile to alter the membrane phospholipid profile [36]. Long chain fatty acid uptake in *E. coli* requires three known proteins, FadL, FadD and PlsB. FadL transports exogenous long chain fatty acids from the environment into the cell where the acyl-CoA synthetase FadD converts the fatty acids into acyl-CoA thioesters and PlsB, an acyl transferase processes them into phosphatidic acid, the precursor for phospholipid synthesis [37], [38], [39]. We identify one of the three homologs of FadL (VC1043) in *V. cholerae* as specifically required for dissemination into the aquatic environment from the host. The other two FadL homologs had neutral phenotypes for all conditions. FadD (VC1985) and PlsB (VC0093) are required for both pathogenesis and dissemination in *V. cholerae*. The identification of FadD, FadL, and PlsB in our screen may highlight the importance of remodeling the lipid composition of the *V. cholerae* membrane during host infection prior to transition into the aquatic environment. This process may be part of a homeoviscous adaption required to survive the membrane stress encountered upon exposure to the host environment and to pond water.

Examination of the fitness values obtained for the genes important for motility revealed a surprising result for infection. The overwhelming majority of flagellar biosynthesis genes and chemotaxis genes are dispensable for infection of the infant rabbit. There exist conflicting results in the infant mouse model for the role of motility and chemotaxis during host infection, while there are some reports that flagellar mutants are attenuated, there are other reports that indicate that flagellar motility is dispensable (Reviewed in [40]). The extensive genome coverage of our data allows us to look at every single gene known to be required for flagellar biosynthesis and chemotaxis, and the results showed that almost all genes in the flagellar biosynthetic and chemotactic pathways show a neutral fitness value in both the small intestine and in the cecal fluid. Flagellar motility is thought to be important for establishing contact with the intestinal epithelium, which induces Cholera toxin mediated secretion of mucus, water, and electrolytes. It is possible that trans complementation by the bulk population (i.e., >99% of population is motile) removes the need for flagellar motility during infection. In this scenario the flagellar motility mutants travel through the gastrointestinal tract without establishing contact with the epithelial cell layer, but may contact the mucus layer and benefit from the environment created by the bulk population. Alternatively, motility may be important under more natural, low dose inoculation scenarios, but this requirement might be obviated following the orogastric inoculation of a large bolus of *V. cholerae*, as was done in this study. In contrast to the *in vivo* environment, there is a requirement for some flagellar biosynthetic genes for full fitness in the aquatic environment. Although the flagellar biosynthetic genes are found in three distinct regions on the *V. cholerae* genome, the majority of the genes important for pond survival are all found at one chromosomal region (VC2187–VC2208) and encode basal body, rod, ring, hook and filament genes, which are parts of the flagellum that have to be secreted through the flagella-specific type three secretion machinery [41]. It is unlikely that the lack of motility in these mutants is causing the survival defect since there are several additional non-motile flagellar mutants identified in our screen that do not have a survival defect in the pond, including important flagellar regulatory genes such as *fliA*, *flrC* and *flrA*. A more plausible explanation is that the survival defect of these flagellar biosynthetic mutants is instead caused by a loss of energy due to misregulation of the remaining flagellar components.

The dissemination of *V. cholerae* from the host into the aquatic environment facilitates the transmission of cholera during epidemics and is also critical for the long-term survival of this pathogen in the environment. Previous studies in the adult rabbit ileal loop model show evidence of a stationary-phase dependent mucosal escape response, where *V. cholerae* detach from the host epithelial cell layer at the late stage of infection when bacterial cell density on the epithelium is high [11]. We therefore explored whether the physiological state of *V. cholerae* affected its ability to disseminate by comparing the survival of exponential-phase, stationary-phase, and host-passaged *V. cholerae* in pond water. The host-passaged and stationary phase bacteria had a significant survival advantage over exponential growth-phase cells. Intriguingly, host-passaged bacteria showed a long-term survival advantage over stationary phase cells.

Using Tn-seq we were able to determine the fitness for 96% of *V. cholerae* non-essential genes during survival in the aquatic environment, resulting in the identification of 165 genes that play significant roles. Our data suggest that in order to survive the transition into the aquatic environment, *V. cholerae* must conserve energy and utilize its current cellular state rather than synthesize anew. For example, we found that several pathways involved in recycling and degradation of amino acids, proteins, and cell wall are important for *V. cholerae* fitness in the pond. *V. cholerae* will likely also need to scavenge nutrients and micronutrients that it can find in the environment, explaining the large number of transporters important for survival in the aquatic environment. One interesting family of transporters identified in our screen is the spermidine/putrescine ABC transporters. These polyamines can bind DNA, RNA and phospholipids and modulate cellular functions [42]. Disruption of *potA*, *potC* and *potD*, all of which are components of the spermidine/putrescine transporter, and *speC*, a putrescine biosynthesis gene all had fitness defects in the aquatic environment. We also identified and validated a set of genes, *mdoG* and *mdoH*, known to be involved in osmoprotection in proteobacteria [43]. These genes are required for periplasmic glucan biosynthesis, which are membrane-derived oligosaccharides that increase during low osmolarity and act as osmoprotectants.

In conjunction with the transcriptional analysis of *V. cholerae* transitioned into pond water from rice water stool [6], we identify a large number of energy production and conversion genes, the phosphate acquisition regulators PhoB and PhoR, along with GlnL and GlnE involved in nitrogen scavenging, to be important for survival in the aquatic environment. Our screen revealed that the two phosphate binding proteins PstS I and II are differentially required in the host and the aquatic environment, although the basis for this differential requirement is not known. Another regulatory system that we identify as important for fitness both in the host and in the aquatic environment but not in rich media is the VarS/VarA two component system. VarS/VarA is homologous to BarA/UvrY in *E. coli*, and has been previously identified in *V. cholerae* to be important for virulence and quorum sensing [23], [26]. We now include a role for VarS/VarA in dissemination. In *E. coli*, BarA/UvrY have been shown to regulate glycogen storage through sRNA inhibition of the carbon storage regulator, CsrA [44]. Glycogen storage granules have been detected in *V. cholerae* isolated from RWS, and some of the glycogen storage and utilization genes have been determined to be important in dissemination [9]. We show that glycogen storage is decreased in a *varS* deletion strain, which can be suppressed by a mutation that decreases the expression of CsrA. These results indicate that VarS/VarA regulates glycogen storage through CsrA in *V. cholerae*, which affects survival in the aquatic environment. We also determined in our screen that the glycogen storage gene *glgA* and the glycogen degradation genes *malQ* and *glgP* are important for survival in both the host and the pond. In contrast, *glgX*, which is needed for breakdown of glycogen stores, is important specifically for pond survival. Glycogen catabolism helps *V. cholerae* meet its carbon and energy requirements during the stressful transition to nutrient-limited aquatic environments. A role for glycogen metabolism during infection of the comparatively nutrient rich intestinal environment is less obvious, but may be related to the integration of glycogen metabolism into central metabolism and bacterial physiology. These results highlight the important role that glycogen storage and catabolism play during the life cycle of *V. cholerae*.

These data provide a comprehensive framework for understanding *V. cholerae* biology during the two critical stages of its life cycle; colonization of the small intestine, and dissemination into the aquatic environment. As such, these data comprise an important resource for future studies into the pathogenesis, dissemination and transmission properties of *V. cholerae*.

## Materials and Methods

### Strains, media and growth conditions

A streptomycin-resistant derivative of *V. cholerae* O1 serogroup, El Tor biotype strain E7946 [45] was used in this study. Single gene knockouts were constructed by utilizing the FRT/FLP recombinase method of deletion as described [22]. *V. cholerae* and *E. coli* were grown on LB agar or in LB broth at 37°C unless otherwise specified. Streptomycin (Sm) was used at 100 µg/ml, Carbenicillin (Carb) at 100 µg/ml, and Kanamycin (Kan) at 100 µg/ml. Strains utilized in this study are shown in Table S5. Primer sequences for gene deletions are listed in Table S6. Pond water was collected from Chandler pond (latitute 42.345223, longitude −71.164262) on 1/9/2011 and Ponkapoag Pond (latitude 42.188406, longitude −71.093241) on 4/7/2011, 9/23/2011, 6/4/2012 and 9/22/2012. Previous analysis determined that the pond water samples had similar chemical composition as those obtained from a cholera endemic area in Southeast Asia [10].

### Transposon library construction

The pUTmTn*5*Km2 plasmid in Sm10λpir was delivered to *V. cholerae* E7946 by mating with selection for mTn*5*Km2 transposition into the recipient genome using similar methods as previously described [20]. In summary, log phase E7946 and Sm10λpir+pUTmTn*5*Km2 were pelleted, resuspended to an OD_600_ = 8.0, and mixed 1∶1. Aliquots of 0.2 ml of the 1∶1 mating mixture were plated onto LB agar plates without antibiotic and incubated for 4 hrs at 37°C. After the 4 hr incubation, the LB plates were replica plated onto Sm100 Kn180 plates and incubated at 37°C for 12 hrs, and then replica plated again on Sm100 Kn180 plates for another 24 hrs at room temperature. Colonies were pooled (11 pools ranging from 5,000–80,000 CFU) and resuspended in LB with 15% glycerol to an OD_600_ of 1.0 and frozen in 1 ml aliquots at −80°C. Library complexity was determined by Tn-seq. Libraries were prepared on two independent days. It is important to note that the mTn*5*Km2 transposon contains factor-independent transcriptional terminators on both transposon ends and therefore disrupts transcription of downstream genes in operons.

### Infant rabbit colonization assays

Litters of 3-day old New Zealand White infant rabbits were obtained from a commercial source (MillBrook Labs, Amherst, MA) and housed together with their doe for the duration of the experiments. The dams and their litters were housed with food and water *ad libitum* and monitored in accordance with the rules of the Department of Laboratory Animal Medicine at Tufts Medical Center. The 3-day old infant rabbits were treated with 300 mg/kg of Cimetidine-HCL (Morton Grove Pharmaceuticals) by oral gavage 3 hrs prior to infection. Rabbits were orogastrically inoculated with ∼5×10^8^ CFU of *V. cholerae*. To prepare the inocula, transposon library aliquots were thawed and diluted 1∶100 in LB containing Sm and Kan and grown at 37°C with aeration until late exponential phase (OD_600_ = 0.5). The bacteria were then pelleted and diluted 1∶2 in LB adjusted to pH 5.7 with HCl and grown for 1 hr at 37°C with aeration. At 1 hr, the acid-tolerized bacteria were pelleted and resuspended in 2.5% sodium bicarbonate buffer (pH 9) to a final concentration of ∼10^9^ CFU/ml. The infant rabbits are euthanized at 12 hr post-infection, when the infant rabbits exhibit signs of secretory diarrhea and Cholera disease. The cecal fluid was collected from the cecum by puncture and the contents were drained into a sterile petri dish. The small intestine was also collected and homogenized in sterile Phosphate Buffered Saline (PBS). A low speed spin (500 RCF) for 2 minutes removed cell debris. Both the cecal fluid and small intestine homogenate was serially diluted and plated on Sm, Km plates for bacterial enumeration. After collection, *in vivo* samples were minimally outgrown in LB by placing 0.1–0.3 ml of either cecal fluid or small intestine homogenate into 8 ml of LB and grown 2–6 hr at 37°C with aeration until turbid (OD_600_ = 1.0). Glycerol was added to a final volume of 20% and 2 ml aliquots were frozen at −80°C until gDNA could be prepped for Tn-seq.

### Tn-seq in rich medium and in pond

Both the culture prior to acid-tolerization and the final inocula used to infect the infant rabbits were diluted and outgrown in LB broth overnight at 30°C (LB and LB-B, respectively). Samples were collected from both the starting population (T1) and the end population (T2) for both enumeration by CFU/ml and for Tn-seq. The LB samples were used to determine the putatively essential genes, the fitness of non-essential genes, and for comparison to gene fitness in pond. The LB-B fitness values were used for comparison to gene fitness in the host.

The overnight stationary phase LB libraries were pelleted, washed twice with filter-sterilized pond water and then diluted 1∶100 in pond water and incubated at 30°C for 48 hrs. Host-passaged *V. cholerae* libraries in the cecal fluid were also placed in pond water for 48 hr at 30°C. First a low speed (500 RCF) spin was used to remove large particulate matter and eukaryotic cells, the supernatant was collected and placed in a new microfuge tube and spun at 12,000 RCF to pellet the bacteria. The pellet was resuspended in 10 ml of pond water and incubated at 30°C for 48 hr. At 48 hr the bacteria were pelleted and resuspended in 8 ml of LB and grown to until OD_600_ = 1.0, aliquoted with glycerol and frozen for Tn-seq.

### DNA sample preparation and Illumina sequencing

The transposon junctions were amplified from gDNA samples and subjected to MPS essentially as previously described [46]. Changes to these methods and primers are specified in the Supplemental Materials and Methods (Text S1).

### Fitness calculation

All read mapping and primary data analysis was done on the Tufts University Galaxy server (http://genomics.med.tufts.edu/home/analysis) using fitness calculation scripts nearly identical to those previously described [15]. Specific details of the fitness calculation for both the Tn-seq screen and the mini-library competition experiment are listed in the Supplemental Materials and Methods (Text S1).

### Competition assays in pond

Overnight stationary phase cultures grown at 37°C with aeration of Δ*lacZ* and either wild type or a deletion strain were mixed 1∶1, serially diluted, and plated on LB agar with 40 µg/ml 5-bromo-4-chloro-3-indolyl-β-D-galactopyranoside (X-gal). A volume of 0.3 ml of the 1∶1 mixture was pelleted by centrifugation and resuspended in 1 ml of filter sterilized pond water. A volume of 10 µl of the resuspended cell mixture was then put in 10 ml of filter sterilized pond water and incubated at 30°C standing for 48 hr. At 48 hr, the tube was vortexed rigorously, serially diluted and plated for CFU/ml on LB X-gal agar plates. The competitive index (CI) was calculated as the ratio of the mutant compared to the control strain normalized to the input ratio. A minimum of five biological replicates was used to calculate the average CI and a one-sample student t-test was used to determine significance.

### Ethics statement

All animal experiments were done in accordance with NIH guidelines, the Animal Welfare Act and US federal law. Tufts University School of Medicine's Institutional Animal Care and Use Committee approved the experimental protocol “B2013-44” that was used for this study. All animals were housed in a centralized and AAALAC-accredited research animal facility that is fully staffed with trained husbandry, technical and veterinary personnel.

## Supporting Information

Table S1 Putatively essential genes in *V. cholerae*.
(XLS)

Table S2 Table of fitness values for all genes in the *V. cholerae* genome.
(XLS)

Table S3 Table of host-specific and pond-specific genes.
(XLS)

Table S4 Table of validation data for deletion strains.
(XLS)

Table S5 Table of strains used in this study.
(DOC)

Table S6 Table of primer sequences used in this study.
(XLS)

Text S1 Supporting materials and methods.
(DOC)
