# Supplementary material for: Gene Fitness Landscapes of Vibrio cholerae at Important Stages of Its Life Cycle
Source: PLoS Pathog. 2013 Dec 26;9(12):e1003800. doi: 10.1371/journal.ppat.1003800 (PMC3873450; doi:10.1371/journal.ppat.1003800)
Supplement: Table S5 — Table of strains used in this study. (DOC) [file ppat.1003800.s005.doc]

**Table S5. Strains used in this study**

| ***V. cholerae* Strains** | **Genotype** | **Reference or Source** |
| --- | --- | --- |
| WT (E7946) | Spontaneous SmR derivative of E7946, El Tor Ogawa | [1] |
| WT + pACtsKan | Ts pSC101 origin, KanR | [2] |
| Δ*lacZ* | In-frame deletion of *lacZ* (VC2338) | [3] |
| Δ*aroA* | In-frame deletion of *aroA* (VC1732) | This study |
| Δ*gltA* | In-frame deletion of *gltA* (VC2092) | This study |
| Δ*dsbA* | In-frame deletion of *dsbA* (VC0034) | This study |
| Δ*nlpD* | In-frame deletion of *nlpD* (VC0533) | This study |
| Δ*degP* | In-frame deletion of *degP* (VC0566) | This study |
| Δ*guaB* | In-frame deletion of *guaB* (VC0767) | This study |
| Δ*cvpA* | In-frame deletion of *cvpA* (VC1003) | This study |
| Δ*yjgF* | In-frame deletion of *yjgF* (VC2512) | This study |
| Δ*ulaR* | In-frame deletion of *ulaR* (VCA0247) | This study |
| Δ*phnR* | In-frame deletion of *phnR* (VCA0600) | This study |
| Δ*mtlD* | In-frame deletion of *mtlD* (VCA1046) | This study |
| Δ*mdoH* | In-frame deletion of *mdoH* (VC1287) | This study |
| Δ*potC* | In-frame deletion of *potC* (VC1426) | This study |
| Δ*ctpA* | In-frame deletion of *ctpA* (VC1496) | This study |
| Δ*ftsX* | In-frame deletion of *ftsX* (VC1629) | This study |
| Δ*fliK* | In-frame deletion of *fliK* (VC2128) | This study |
| Δ*flgD* | In-frame deletion of *flgD* (VC2198) | This study |
| Δ*ampG* | In-frame deletion of *ampG* (VC2300) | This study |
| Δ*rimK* | In-frame deletion of *rimK* (VC2333) | This study |
| Δ*cmaX* | In-frame deletion of *cmaX* (VC2334) | This study |
| Δ*ampD* | In-frame deletion of *ampD* (VC2421) | This study |
| ΔVC2542 | In-frame deletion of VC2542 | This study |
| Δ*ldtB* | In-frame deletion of *ldtB* (VCA0058) | This study |
| ΔVCA0762 | In-frame deletion of VCA0762 | This study |
| ΔVCA0999 | In-frame deletion of VCA0999 | This study |
| Δ*miaA* | In-frame deletion of *miaA* (VC0346) | This study |
| Δ*hapR* | In-frame deletion of *hapR* (VC0583) | This study |
| Δ*corC* | In-frame deletion of *corC* (VC0959) | This study |
| Δ*rsuA* | In-frame deletion of *rsuA* (VC1179) | This study |
| Δ*vceR* | In-frame deletion of *vceR* (VC1408) | This study |
| ΔVC1444 | In-frame deletion of VC1444 | This study |
| ΔVC1807 | In-frame deletion of VC1807 | This study |
| Δ*ygbF* | In-frame deletion of *ygbF* (VC1834) | This study |
| Δ*vanY* | In-frame deletion of *vanY* (VC2153) | This study |
| Δ*leuO* | In-frame deletion of *leuO* (VC2485) | This study |
| Δ*varS* | In-frame deletion of *varS* (VC2453) | This study |
| ∆*varS*** | In-frame deletion of *varS* (VC2453) and a G→A SNP at 580591. | This study |
| ***E. coli* Strains** | **Genotype** | **Reference or Source** |
| SM10λpir+ pUTmTn5Km2 | AmpR plasmid pUTmTn5Km2 containing mTn5 transposon with KanR cassette in | [4] |

**References:**

1. Levine MM, Black RE, Clements ML, Cisneros L, Saah A, et al. (1982) The pathogenicity of nonenterotoxigenic *Vibrio cholerae* serogroup O1 biotype El Tor isolated from sewage water in Brazil. J Infect Dis 145: 296-299.

2. Tamayo R, Patimalla B, Camilli A (2010) Growth in a biofilm induces a hyperinfectious phenotype in *Vibrio cholerae.* Infect Immun 78: 3560-3569.

3. Bradley ES, Bodi K, Ismail AM, Camilli A (2011) A genome-wide approach to discovery of small RNAs involved in regulation of virulence in *Vibrio cholerae.* PLoS Pathog 7: e1002126.

4. Merrell DS, Hava DL, Camilli A (2002) Identification of novel factors involved in colonization and acid tolerance of *Vibrio cholerae.* Mol Microbiol 43: 1471-1491.
