# Supplementary material for: Gene Fitness Landscapes of Vibrio cholerae at Important Stages of Its Life Cycle
Source: PLoS Pathog. 2013 Dec 26;9(12):e1003800. doi: 10.1371/journal.ppat.1003800 (PMC3873450; doi:10.1371/journal.ppat.1003800)
Supplement: Text S1 — Supporting materials and methods. (DOC) [file ppat.1003800.s007.doc]

**Text S1 : Supplementary Materials and Methods**

**Sample Preparation and Illumina Sequencing**

Samples were prepared using similar methods as previously described [1]. In summary, gDNA was prepared from frozen bacterial pellets using a DNeasy (Qiagen) kit. The gDNA was then sheared by high intensity sonication and a poly-C tail was added to the 3’ end using a Terminal deoxynucleotidyl Transferase. Two PCR reactions were used to amplify the gDNA-Tn junctions and to attach specific sequences required for Illumina sequencing and indexing. The first PCR uses a poly-C specific (olj376) and a transposon specific (olj491) primer pair. The second PCR uses one of several indexing primers (5’CAAGCAGAAGACGGCATACGAGATNNNNNN

GTGACTGGAGTTCAGACGTGTGCTCTTCCGATCT 3’, where NNNNNN represents the reverse complement of the index and varied with each sample) and a transposon end-specific primer (olj492). Libraries were then pooled and run in a single end sequencing reaction on an Illumina Genome Analyzer II (Tufts University) using the custom sequencing primer HK89.

For the mini-TN-seq validation, olj376 was paired with HK178 in the first PCR reaction and HK179 was paired with an indexing primer in the second PCR reaction. The custom sequencing primer HK180 was used.

**Fitness Calculation**

All read mapping and primary data analysis was done on the Tufts University Galaxy server using fitness calculation scripts nearly identical to those previously described [2]. All sequences were trimmed to 35 bp in length and then mapped to the *V. cholerae* N16961 genome with the program Bowtie. Bowtie parameters were set to only allow 1 mismatch (-n 1) in the seed and (-best) guarantee that alignments are ‘best’ at the mismatched position. In the fitness calculation, we required a minimum of 20 reads in T1 and (T1+T2)/2 must be >15 reads. Insertion sites in the last 10% of each gene were excluded as well as sites in intergenic regions. Expansion factor (d) was calculated as previously described by plating CFU/ml at both T1 and T2 and included the population expansion during LB outgrowth*.* To determine the expansion factor during host infection we utilized pACtsKan, a temperature-sensitive plasmid that is replication defective at 37°C. We determined a standard curve of plasmid loss during expansion *in vitro* at 39°C by enumerating bacteria on both Sm and Sm plus Kan plates (Figures S1). The same strain was then used to infect six 3-day old infant rabbits per the standard protocol. At 12 hrs post-infection the rabbits were sacrificed and cecal fluid and small intestine were collected and processed. The *V. cholerae* was enumerated on both Sm and Sm plus Kan plates from both the cecal fluid and the small intestine. By fitting the ratio of plasmid loss *in vivo* to the *in vitro* standard curve, the expansion of the *in vivo* population was estimated and used in our fitness calculation.

After fitness was determined, the data was normalized against a set of neutral insertion sites found in these genes (VC0068, VC0153, VC0484, VC0818, VC1807, VC2497, VCA0358, VCA0449, VCA0454), which contain authentic frameshift mutations [3]. Stochastic loss of mutants due to bottleneck effects was calculated based on the loss of insertions in our neutral gene list. The bottleneck was then applied to all genes individually by removing the same proportion of lost insertion sites as previously described [2,4]. All insertions in each gene across all biological replicates were aggregated to calculate a gene’s average fitness and standard deviation. Genes were listed as “putatively essential” if 1) the gene had a fitness of zero in LB or 2) <2 insertion sites were detected in LB and the gene size is >350bp. However, if there are <2 insertion sites in LB but there is a non-zero fitness value in either pond or host then that gene is excluded from the list.

To determine whether a gene fitness significantly differed from wild-type (1.0), the fitness had to be calculated from ≥5 insertions and the fitness had to be significantly different than 1.0 in a one sample t-test with Bonferroni correction (*P* value <0.000015). To determine if a fitness value was specific for the selective condition, we required the fitness value to be statistically different than the fitness value obtained in LB by applying a student's t-test using Bonferroni correction and required at least a 0.15 fitness value difference between the two fitness values. Any gene with a fitness value less than 0.82 in LB was determined to have a general growth defect and eliminated from the host and pond specific gene lists.

**Mini-Tn-Seq Validation**

The 35 deletion strains were grown in LB to mid-log and then mixed equally proportional, pelleted by centrifugation, resuspended in LB + 20% glycerol to an OD_600_= 1.0, aliquoted and frozen at -80˚C. The pre-mixed aliquot was thawed and prepared using the same methods as the original Tn-seq library.

**Infant mouse mini-Tn-seq competition**

Mini-Tn-seq competition experiment was done using 4-5 day old CD-1 mice. The dams and their litters were housed with food and water *ad libitum* and monitored in accordance with the rules of the Department of Laboratory Animal Medicine at Tufts Medical Center. The inoculum was prepared from the premixed frozen stock of 35 deletions strains diluted 1:100 and grown to mid-log. Mice were infected intragastrically with ~10^5^ bacteria and sacrificed 24 hr post-infection. Small intestines were homogenized in LB + 15% glycerol, serially diluted and plated on LB + Sm agar plates for enumeration. 50 μl of the mouse homogenate was diluted in 8ml of LB + Sm and grown 4-6 hr at 37˚C. Glycerol was added to a final volume of 20% and 2 ml aliquots were frozen at -80C until gDNA could be prepped.

Samples from the inoculum (input) and the homogenate (output) were prepared for MPS and mapped to the *V. cholerae* genome as described above. The bowtie output file was then used as input for the “hopcount” script as previously described [1]. The neutral gene VC1807 was used to normalized the reads. The normalized output was divided by the normalized input to generate a Competitive Index (CI) for each deletion in each mouse. One litter of 8 mice were used to generate a median CI for each deletion and a Wilcoxon signed-rank test was used to determine if the Median was statistically different than 1.0.

**Glycogen quantification**

Glycogen content in each strain was determined as previously reported by digesting with amyloglucosidase followed by quantification of released glucose using tetrazolium blue reagent [5] with the following protocol modifications. Strains were patched on Kornberg agar plates (1.1% K_2_HPO_4_, 0.85% KH_2_PO_4_, 0.6% yeast extract, 1% glucose) and incubated overnight at 37˚C. Bacteria were scraped into 1 ml of phosphate-buffered saline (PBS) and adjusted to a final OD_600_ of 5.0 [~5x10^9^ colony forming units (cfu) ml^-1^], pelleted, re-suspended in 1 ml of 50 mM Sodium Acetate pH 4.8 and placed in a screw cap o-ring tube containing 500 μl of 0.1 mm zirconia beads. Samples were processed in a BeadBeater (Biospec Products, inc.) for 2 minutes 45 seconds, after which 500 μl of the processed supernatant was removed to a new microfuge tube to which 20 units of amyloglucosidase (Sigma 10113) was added and incubated overnight at 57˚C in a heat block with a heated lid. The next day the samples were clarified by centrifugation prior to adding the supernatant to the tetrazolium blue reagent as previously described [5]. The absorbance of each sample was compared with a standard curve generated with known concentrations of glycogen from bovine liver, type IX (sigma) that was treated identically in order to determine the concentration of reducing sugars (reducing equivalents) released following amyloglucosidase digestion in each sample. The control sample was processed identically to all other samples except the amyloglucosidase was not added.

**Supplemental References**

1. Klein BA, Tenorio EL, Lazinski DW, Camilli A, Duncan MJ, et al. (2012) Identification of essential genes of the periodontal pathogen *Porphyromonas gingivalis*. BMC Genomics 13: 578.

2. van Opijnen T, Bodi KL, Camilli A (2009) Tn-seq: high-throughput parallel sequencing for fitness and genetic interaction studies in microorganisms. Nat Methods 6: 767-772.

3. Rolfs A, Montor WR, Yoon SS, Hu Y, Bhullar B, et al. (2008) Production and sequence validation of a complete full length ORF collection for the pathogenic bacterium *Vibrio cholerae*. Proc Natl Acad Sci U S A 105: 4364-4369.

4. van Opijnen T, Camilli A (2010) Genome-wide fitness and genetic interactions determined by Tn-seq, a high-throughput massively parallel sequencing method for microorganisms. Curr Protoc Microbiol Chapter 1: Unit1E 3.

5. Bourassa L, Camilli A (2009) Glycogen contributes to the environmental persistence and transmission of *Vibrio cholerae*. Mol Microbiol 72: 124-138.
